# Puerperal hematoma: a cause of post partum hemorrhage after a normal vaginal delivery

**DOI:** 10.11604/pamj.2015.20.365.6478

**Published:** 2015-04-14

**Authors:** Adil Elghanmi, Hicham Seffar

**Affiliations:** 1 Faculté de Médecine de Rabat, Maroc

**Keywords:** Puerperal hematoma, post partum, hemorrhage, vaginal delivery

## Image in medicine

A 28 year old Moroccan woman Para 1 Gravida 1 with no medical or surgical history was admitted to our Obstetric Department of Maternité de Souissi, Rabat, Morocco. She delivered vaginally of a healthy girl of 3600 grams after vacuum extraction and episiotomy. Two hours later, she developed signs of shock. She had a pulse of 120 beat per minute and a blood pressure of 80/60 mmHg. On physical exam, the abdomen was tender, the uterus was tonic. Perineal exam revealed an important right vulvo-vaginal hematoma of approximately 10 centimeters in diameter. Immediately, intravenous access was established and plasma expenders and oxygen were given. A blood test revealed a low hemoglobin rate of 6.3g/dl.3 blood packs were given to her. In the operating theater and under general anesthesia, the puerperal hematoma was drained. The origin of the bleeding was located in the right vaginal wall and in the ischio-rectal fossa. It consisted of venous and arterial rupture of branches of the vaginal and internal pudendal arteries. Hemostatic sutures were performed and different vaginal fascia and perineal tissues were closed. A packing was left in the vagina with compressive wound dressing. Six hours later, the packing was removed with no signs of active bleeding. The patient was given prophylactic antibiotic and pain killers. The patient was released home 2 days after her admission. The follow-ups, one at one week and another at3 weeks were both uneventful.

**Figure 1:**
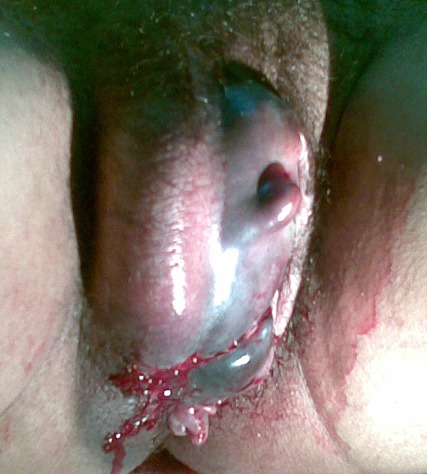
Vulvovaginal hematoma

